# Implementation of the four habits model in intermediate care services in Norway: a process evaluation

**DOI:** 10.1186/s12913-024-11647-z

**Published:** 2024-10-08

**Authors:** Linda Aimée Hartford Kvæl, Pål Gulbrandsen, Anne Werner, Astrid Bergland

**Affiliations:** 1 Department of Rehabilitation Science and Health Technology, Faculty of Health Sciences, OsloMet - Oslo Metropolitan University, Oslo, Norway; 2 Department of Ageing Research and Housing Studies, Norwegian Social Research - NOVA, OsloMet - Oslo Metropolitan University, Oslo, Norway; 3 Institute of Clinical Medicine, University of Oslo, Oslo, Norway; 4 Health Services Research Unit (HØKH), Akershus University Hospital, P.O. Box 1000, Lørenskog, 1478 Norway

**Keywords:** Implementation, Process evaluation, The Four habits Model, Intermediate Care, Clinical communication, Simulation, Normalisation process theory, Norway

## Abstract

**Background:**

Intermediate care (IC) services bridge the transition for older patients from the hospital to the home. Despite the goal of involving individuals in their recovery process, these services often become standardised, leading to communication breakdowns. While evidence-based practices, such as the Four Habits Model (4HM), for effective communication are crucial for enhancing high-quality healthcare, research suggests their integration into routine practice remains limited. In this study, we aimed to investigate the implementation process of the 4HM through a two-day course that engaged healthcare professionals and managers in IC.

**Methods:**

We conducted a process evaluation employing qualitative and quantitative methods: (i) individual interviews with three managers and two course participants pre-course, (ii) two focus group interviews with course participants (*N* = 11) and individual interviews with the same three managers post-course, and (iii) the NoMAD questionnaire (Normalisation MeAsure Development) administered four months later to assess the short- and long-term impact on course participants (*N* = 14). Reflexive thematic analyses were guided by Normalisation Process Theory (NPT), which offers insight into how new interventions become routine practices. The analysis of the NoMAD involved descriptive statistics.

**Results:**

We identified four themes in the qualitative data: (i) Decoding Interactions: Making Sense of the 4HM in IC services, (ii) Fostering Change: Legitimising 4HM Through Staff Engagement, (iii) Harmonising Practice: Integrating 4HM into Complex Situations, and (iv) Embedding Value: Normalising the 4HM into Everyday Work. These themes illustrate the normalisation process of the 4HM course within IC, establishing standard practices. Healthcare professionals and managers highlighted the urgent need to integrate communication skills based on the 4HM into daily care. They noted positive changes in their communication habits following the course. The consistent findings from the NoMAD questionnaire underscore the sustainability of implementing the 4HM programme, as participants continue to utilise it in their clinical practice beyond the initial four-month period.

**Conclusion:**

The 4HM course programme was deemed feasible for expansion within IC services. Both managers and staff found its focus on addressing communication breakdowns and readiness for change sensible. The study findings may benefit the stakeholders involved in IC service routines, potentially improving services for older patients and relatives.

**Supplementary Information:**

The online version contains supplementary material available at 10.1186/s12913-024-11647-z.

## Contributions to the literature

- This pioneering study investigates the implementation of the Four Habits Model (4HM) through a two-day course programme for intermediate care (IC) services, offering valuable insights for scaling up in similar municipal contexts.
- Healthcare professionals and managers found the 4HM course feasible, with motivated participants, skilled instructors, and supportive managers aiding its integration, while long-term success relies on systematic Train-The-Trainer education for sustained improvement in IC communication.
- Applying normalisation process theory to illuminate the acceptance and integration of the 4HM course programme into IC services contributes new knowledge to the field, illustrating effective implementation strategies in healthcare contexts.

## Background

Supporting the growing popularity of ageing in place [1], intermediate care (IC) services aim to support transitional care for older patients, from the hospital to the home [2]. These IC services can be provided in special hospital units, in nursing homes, in the patients’ homes or as short-term, institution-based municipal rehabilitation [3]. Grounded in a rehabilitative philosophy, IC services are designed to facilitate patient participation aligned with the individualised needs of patients and their relatives [4]. Despite aiming for patient participation in recovery and restoring independence, these services frequently become standardised, leading to communication breakdowns [5–7]. Communication competence is therefore essential to ensuring that IC services meet the individual needs of patients. In this regard, the World Health Organization recommends interprofessional healthcare education as a key strategy to address communication challenges [8, 9].

Efficient communication can influence patient satisfaction [10], the frequency of complaints [11], patient compliance [12], healthcare service utilisation [13] and overall patient outcomes [14, 15]. Given its importance, implementation research has increasingly focused on strategies to embed effective practices in healthcare settings [16]. According to Proctor et al. (2013), *implementation strategies* are defined as ‘methods or techniques used to enhance the adoption, implementation and sustainability of a clinical programme or practice’ [17, p. 2]. One such strategy is the Four Habits Model (4HM) communication course designed to enhance interactions between healthcare professionals and patients [18]. Although the 4HM course model is used in specialist healthcare services in Norway, its effectiveness in primary care settings has not been tested. In April 2023, we introduced the 4HM in municipal IC services. Their experiences were described in a previous study [19]. This paper focuses on the implementation process of the 4HM in IC.

### The four habits model

The Four Habits Model (4HM) is a communication model developed in the United States by Frankel and Stein (1999); it was originally designed for physicians’ use in routine practice. The model comprises four trainable elements: (i) invest in the beginning through building trust, (ii) elicit the patient’s perspective, (iii) demonstrate empathy and (iv) invest in the end [18]. The effectiveness of the 4HM in interactions with general practitioners (GPs), hospital doctors [20–23] and nurses [24, 25], have been extensively documented and validated. However, another nursing study did not observe a notable shift in empathy [26]. The 4HM has shown effectiveness in supporting patients experiencing emotional distress [27] and holds promise, as indicated in a recent qualitative study during IC family meetings [28].

Implementation strategies, such as the 4HM course programme, must be tailored to the various phases of implementation [29]. According to the Exploration, Planning, Implementation and Sustainability (EPIS) framework, the implementation process comprises distinct phases that must be considered, along with inner and outer contextual characteristics, to bridge these two contexts and the characteristics of the intervention to be implemented [30]. Along with this, the active participation of stakeholders is considered essential for the successful implementation of knowledge. However, many approaches lack clear specifications and validation [16]. Nonetheless, research conducted among persons living with multimorbidity, the typical IC patient, highlights that effective interprofessional communication involving patients is crucial for improving their everyday lives [31, 32].

As is the case in this study, there is growing emphasis on patients’ and users’ rights to participate in research [33], i.e., engaging users in setting research priorities, which can bridge the gap between patient needs and research agendas, thus minimising research waste [34]. When users participate in setting research priorities, they gain a stronger sense of owning the research and its outcomes. This may influence the implementation of the research results, as the research is perceived as more relevant [35]. The James Lind Alliance (JLA) process is one method that identifies and priorities unanswered questions or evidence-related uncertainties that relevant stakeholders, such as staff, patients, and relatives, agree are most important [36].

### Normalisation process theory

The implementation of complex interventions, such as the 4HM course programme, demands attention to the social processes needed to integrate the intervention into daily practice [37]. Normalisation Process Theory (NPT) addresses crucial factors essential for the successful implementation and integration of interventions into routine work. NPT is a sociological theory that targets three core issues: implementation (bringing the practice into action), embedding (incorporating the practice into daily routines) and integration (ensuring the sustainability of the new practice) [37]. The NPT framework provides researchers with an approach to describing, evaluating and enhancing the potential for successful implementation [38]. The theory proposes four mechanisms to elucidate the process of how new practices become integrated into regular routines: coherence, cognitive participation, collective action and reflexive monitoring [37]. NPT has guided qualitative and quantitative analyses across several healthcare settings and informed implementation processes [39–41]. To the best of our knowledge, NPT has not been utilised in the IC context as a framework for exploring the implementation of the 4HM.

### Study aim

In this study, we aimed to investigate the implementation process of the 4HM through a two-day course that engaged healthcare professionals and managers in IC services.

## Methods

### Design

Based on a JLA process and from a pragmatist perspective [42], we employed a process evaluation design [43] using NPT [37]. This design included both qualitative and quantitative methods: (i) individual interviews with three managers and two course participants pre-course, (ii) two focus group interviews with course participants (*N* = 11) and individual interviews with the same three managers post-course, and (iii) the administration of the NoMAD questionnaire four months later to assess the short- and long-term impact among course participants (*N* = 14).

### The 4HM course programme in IC

The Nursing Home Agency in the capital of Norway, Oslo, oversees four IC institutions, facilitating the transition from specialised to primary care through short-term rehabilitation by interdisciplinary teams [2]. This study was conducted in one of these IC institutions with 142 beds. IC is characterised by a high turnover of patients, placing significant demands on effective communication skills between healthcare professionals, patients and their relatives. The 4HM course programme, focusing on the four key habits, aims to enhance patient satisfaction, improve treatment compliance and reduce complaints [20]. The habits are interconnected sequentially, forming a state of interdependence, each corresponding to a set of communication skills [18].

The 4HM programme includes plenary sessions and role-playing exercises. Interprofessional simulation is an active implementation strategy that demonstrates the potential for improving clinical communication to promote patient participation in transitional care [44, 45]. Instructors explain the simulation process and provide feedback forms to foster a safe atmosphere. Participants engaged in role play as healthcare staff and patients or relatives. Case scenarios from the IC context were selected to ensure authenticity. With funding support, we released healthcare professionals from clinical practice for two full days to attend the course programme without disruptions. The instructors, including the first author and an external researcher, are both 4HM certified and have backgrounds as former physiotherapists with clinical experience in IC services. The first author consulted experts in the research field and had access to educational materials. Adjustments were made to the course’s content and focus based on experts’ opinion while adhering to the original template structure to cater to the specific needs of the target audience, namely, healthcare professionals in IC. Context-specific educational materials were developed, such as a film depicting real-life IC scenarios, information handouts and an IC pocket card illustrating the four habits.

### Recruitment

In autumn 2022, the first author presented the evidence-based nature of the 4HM course to the leadership group, identifying suitable wards within the IC institution. In April 2023, two courses were organised with seven and eight participants, respectively. Ward managers disseminated information, enlisted volunteer healthcare professionals and facilitated the courses. The course participants, a strategic sample, included 15 healthcare professionals with diverse professional backgrounds and experiences from four wards in the IC institution. They were motivated staff members who served as resource persons. The idea was that individuals who are highly engaged and proactive can influence the attitudes and behaviours of their colleagues. Additionally, three managers participated: a department manager with personnel responsibility, a nurse responsible for cross-departmental professional development and a quality manager with both professional and personnel responsibilities. Thus, the total number of participants, including managers and course participants, was 18 (*N* = 18).

### Data collection

**Fig. 1:**
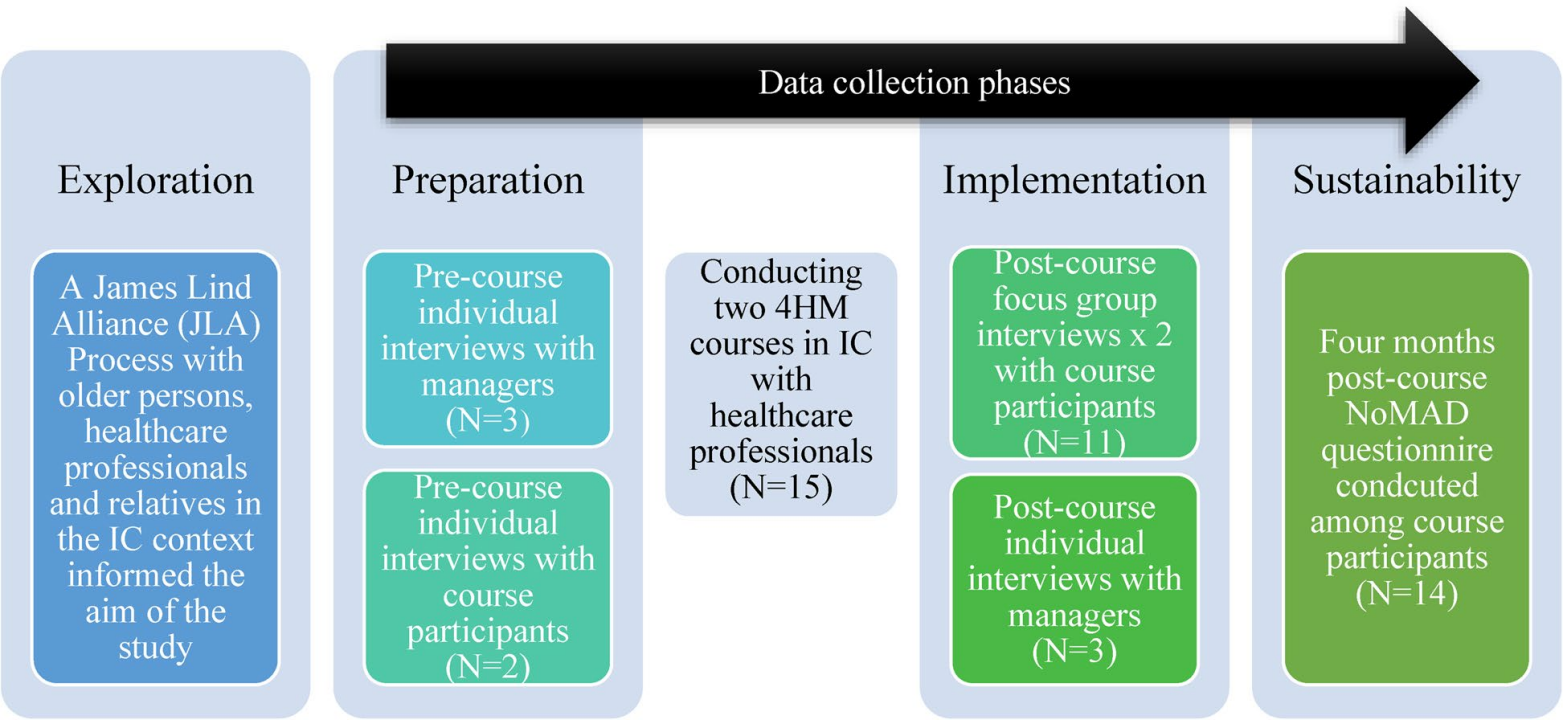
Overview of 4HM implementation process with data collection phases

During the exploration phase, we conducted a pre-study guided by the JLA guidelines for involving patients, and staff to identify and prioritise needs for healthcare development [36]. The top priority identified was ‘enhancing knowledge and awareness to improve patient participation and communication in IC services’. This informed our study’s aim to evaluate the implementation process of the 4HM into IC through a two-day communication course. Figure 1 provides an overview of the 4HM process of implementation in accordance with the EPIS framework, highlighting the study’s data collection phases.

In the preparation phase, the first author conducted five individual semi-structured interviews (three managers and two course participants) to assess the readiness for introducing the 4HM course programme into IC services. The interviews lasted between 45 and 60 min. Interview topics covered knowledge about user involvement, effective communication, measures and leadership support, ward climate, acceptance, implementation of the 4HM course and strategies for sustaining practice changes (see pre-course interview guide in Supplementary Material 1).

During the implementation phase, two to four weeks after the 4HM courses attended by 15 staff members, we conducted two focus group interviews with 11 of these participants to explore their experiences with the course. These interviews were led by an external researcher not involved in the intervention while observed by the last author and lasted between 75 and 90 min. To ensure information power [46], the first author conducted follow-up interviews with the same three managers (duration 30–40 min) who had not participated in the courses but were actively involved in their preparation and maintained frequent dialogue with the 15 course participants (*N* = 18). The post-course focus group and individual interview guides can be found in Supplementary Material 2. All interviews were recorded as MP3 files and transcribed verbatim by the first author.

During the sustainability phase, four months later, 14 of 15 course participants completed the NPT-based NoMAD questionnaire [47]. This questionnaire captures the perspective of healthcare professionals actively engaged in the implementation of complex interventions within the healthcare setting to understand the dynamics of implementing, embedding and integrating new practices, such as the 4HM. It comprises 23 items, including 20 items rated on a 5-point scale (ranging from completely agree to completely disagree) and three items rated on a 1–10 scale. Higher scores indicate greater favourability towards normalisation.

The NoMAD tool shows strong face validity, construct validity and internal consistency when evaluating staff perceptions regarding factors pertinent to integrating interventions that alter their work practices [48, 49]. The NoMAD questionnaire is provided as Supplementary Material 3.

### Data analysis

To analyse qualitative material, we performed an experientially oriented reflexive thematic analysis, a six-phase process starting with the familiarisation of data and moving into a systematic coding process before exploring, developing, reviewing and refining themes [50].

To familiarise with the data, the first author thoroughly reviewed the transcripts to ensure accuracy, anonymisation and alignment with the original audio files while taking notes. Pre-interviews, focus group and post-interviews were combined and organised into one dataset and inductively coded by the same author using HyperResearch software version No. 4.5.3. The goal was to identify patterns across the data, targeting the implementation of the 4HM. An example of the coding process is provided in Table 1.

**Table 1:** The coding process, from quotations to initial themes

Quotes about the implementation of the 4HM	Codes	Initial theme
*‘When implementing new routines*,* we need time to learn them. Yes*,* time and training. Some can learn quickly. For us*,* who are a bit older*,* it takes a little longer. For example*,* we used a fall screening tool. Now we have something new to learn*,* but why has it changed …*,* we don’t know’.* (No. 6, pre-interview course participant)	Need for time and training Understanding the change	Making sense of the 4HM routine
‘*Simulation or role-playing activities can feel intimidating and unfamiliar for many individuals*,* as it’s not a format we’re accustomed to. We’re healthcare professionals*,* not actors. Nevertheless*,* I think active participation in role playing is far more effective than merely observing staged scenarios’*. (No. 4, focus group 1)	Simulation can be scary Active strategies are efficient for learning	Engagement of healthcare professionals

Implementation patterns were categorised into initial themes, including understanding a new routine, simulation as an efficient learning method, engaging staff and managers, leadership anchoring, integrating the 4HM into complex daily IC care and strategies for lasting change.

Accordingly, at this point, the NPT was used as a theoretical lens for further analysis to investigate facilitators and barriers influencing the implementation of the 4HM course programme. This collaborative process between the first and last authors involved thematic mapping, reviewing and naming the themes to uncover both their apparent significance and underlying essence in the implementation process of the 4HM. Based on prior experience, the authors conducting the analysis were aware of their preunderstanding that improving communication competence in the IC could be challenging. The final analysis resulted in four main themes presented in the Results section. The primary author then summarised the contents of the main themes and identified illustrative quotations in a recursive process.

The analysis of the NoMAD questionnaire involved descriptive statistics, including frequencies and medians. Data were assessed using the Statistical Package for the Social Sciences (SPSS) Version No. 28. As the NoMAD Questionnaire corresponds with the NPT, the survey’s results will be illustrated, compared and complemented with the qualitative analysis in the Discussion section.

## Results

The Results section will first provide an overview of the participants’ characteristics, then present the qualitative analysis, followed by the findings from the NoMAD questionnaire.

### Participants

Among the 18 participants (seven nurse assistants, five registered nurses, three therapists and three managers), all were female except one. Their ages ranged from 24 to 58 years, with an average of 43 years, and they had, on average, more than 13 years of healthcare experience. Table 2 provides an overview of the participants’ characteristics and data contributions.

**Table 2:** Characteristics of the study’s participants (*N* = 18)

**Age in years**	***n = 18***
<30	4
30–39	2
40–49	5
50–59	7
**Sex**	***n = 18***
Male	1
Female	17
**Profession**	***n = 18***
Registered nurse	5
Nursing assistant	7
Therapists	3
Manager	3
**Experience in healthcare (in years)**	***n = 18***
0–5	4
6–15	8
16–25	4
26–30	2
**Length of experience with IC (in years)**	***n = 18***
<1	3
2–5	6
>5	9
**Ward**	***n = 15*** ^a^
1	4
2	6
3	4
4	1

^a^Applies to the course participants only, not mangers

### Results from the qualitative analysis

The main themes identified from the qualitative analysis were (i) Decoding Interactions: Making Sense of the 4HM in IC services, (ii) Fostering Change: Legitimising 4HM Through Staff Engagement, (iii) Harmonising Practice: Integrating 4HM into Complex Situations and (iv) Embedding Value: Normalising the 4HM into Everyday Work (Table 3). The four themes encompass and form the normalisation process of the 4HM course programme, which involves establishing and adhering to standard practices or actions within IC services and as such represent different aspect of the implementation process. The four themes, which are interrelated and dynamic rather than static or isolated, represent a process that reflects the complexities and variations involved in evaluating implementation processes.

**Table 3:** The results of the analysis

Themes	Characteristics of the themes
Decoding Interactions: Making Sense of the 4HM in IC services	Strengthening communication and involving users in IC are crucial steps for making progress
	Highlighting the vital role of leadership support in the anchoring of the 4HM course programme
	The importance of introducing and contextualising is pivotal for ensuring a clear understanding of the 4HM
Fostering Change: Legitimising 4HM Through Staff Engagement	Accelerating success by obtaining funding and involving motivated staff to streamline processes
	Instructors with clinical experience with IC services and Train-The-Trainer (TTT) Certification for 4HM course
	Utilising simulation as an active and effective learning method applicable to real-world practice scenarios
Harmonising Practice: Integrating 4HM into Complex Situations	Empowering leaders should actively engage, monitor progress and facilitate space for 4HM initiatives
	The 4HM is applicable in all patient and family interactions, as well as among the IC team members
	Addressing barriers such as fragile patients, high patient turnover and limited professional autonomy
Embedding Value: Normalising the 4HM in Everyday Work	There is a consensus that effective communication enhances patient satisfaction and reduces complaints
	IC staff have adjusted their communication practices following their completion of the 4HM course
	Due to positive feedback, the Nursing Home Agency will implement the 4HM on a full scale through TTT

#### Decoding interactions: making sense of the 4HM in IC services

In the first theme, the participants stressed the importance of understanding ordinary clinical encounters to effectively apply the 4HM in IC service delivery.

During the pre-interviews in the preparation phase, participants noted the significant need for healthcare professionals to integrate communication skills into daily care. They noted a disconnect between procedures, patient involvement and information. This lack of integration was identified as the main cause of patient complaints, which were particularly noticeable during patient arrivals. The informants emphasised the urgent need to improve communication and patient involvement in IC, indicating their readiness for change.

‘*When we receive complaints*,* they usually revolve around user involvement or the adequacy of information we have provided. I believe that many staff members do not connect user involvement with communication. We tend to be very focused on tasks and templates*,* overlooking the inclusion of patients and their relatives*,* such as how we interact with patients*,* our attitudes and our responsiveness to their inquiries. So*,* I believe we tend to view these aspects separately*,* even though they should be integrated*’. (No. 16, pre-interview manager)

Managers also highlighted this knowledge gap within the leadership group when introducing the 4HM course programme to IC services. They affirmed that effective communication and user involvement align with IC values and their rehabilitative philosophy. They viewed the programme and project as well organised and firmly established at the directorial level. This provided department heads with a strong foundation to inform and recruit participants, extending compelling invitations.

‘*My manager*,* with whom I’ve worked for 15 years*,* expressed interest in attending the course herself. I thought it commendable that she also wanted to participate. Consequently*,* she strongly recommended it to me*’. (No. 1, focus group interview 2)

Both course participants and managers emphasised the importance of comprehending the potential benefits of the 4HM in clinical practice, distinguishing it from current approaches. They found the learning materials, including pocket notecards, relevant and well balanced with theory and active learning formats. Presenting 4HM as evidence-based practice, rooted in high-quality research and contextualising it with real-life scenarios within IC services, made the course programme relevant. Participants noted that healthcare professionals in IC often experience fatigue from constantly adopting new procedures. Thus, integrating new routines seamlessly into practices and providing a tool to navigate clinical encounters and deliver higher-quality care were perceived as a relief in subjects’ busy workday.

‘*We tend to operate on autopilot throughout the working day. The 4HM course acted as a refresher for many of us*,* encouraging us to reflect on how we can systematise our patient approach and be more conscious of our actions*,* which are often carried out in a somewhat disorderly manner*’. (No. 12, focus group interview 2)

Healthcare professionals and the management group found the 4HM sensible due to a significant gap in the staff’s understanding and awareness of communication breakdowns, coupled with a readiness for change.

#### Fostering change: legitimising 4HM through staff engagement

Theme 2 promotes change by engaging staff in simulation exercises guided by trained instructors to secure acceptance and support for the 4HM.

Based on pre-interviews, we formed two 4HM courses, dividing a diverse group of members from four departments to ensure a mix of experience and fresh insights. Informants suggested inviting motivated IC staff, especially those who could serve as role models, to kickstart the programme and inspire others to join, validating the utility of the 4HM in IC.

*‘At first*,* I considered inviting people with limited communication skills to participate in the course*,* but that appeared to offer only short-term benefit. I came to understand that what we truly need are “super users”*,* individuals who are highly motivated*,* skilled and capable of effectively promoting the 4HM. These super users can bring back their knowledge and inspire other staff in the ward*’. (No. 18, pre-interview manager)

Course participants stressed the need for skilled instructors with practical experience and strong communication expertise. They felt such instructors fostered a safe learning environment by empathising with staff, understanding their busy schedules and recognising relevant practice situations. Subjects highlighted that demonstrating simulations first and offering feedback were reassuring methods.

‘*I valued their clinical experience and the role-playing demonstrations provided. It helped alleviate the fear of making mistakes. The instructors initially focused on offering constructive praise*,* followed by thoughtful criticism. They stressed the importance of acknowledging what was done well and fostering a supportive atmosphere. In instances where things didn’t go as expected*,* there was space to lighten the mood and take a break*,* which helped reduce anxiety*’. (No. 14, focus group interview 1)

Course participants felt appreciated to be relieved from clinical duties for two days to attend the 4HM course, acknowledging the opportunity. They found simulation, an interactive learning method, the most effective for practising communication skills, especially in real-life situations. Despite some challenges and discomfort, tailored role-playing scenarios felt genuine and beneficial to participants.

‘*I learned a lot*,* even though I find it scary to simulate role playing. However*,* I also think it was a very good combination of theory. It wasn’t too much theory*,* with simulation*,* and the fact that you could provide feedback. And it was a small group*,* so it wasn’t so awkward and scary*’. (No. 10, focus group interview 1)

Prioritising motivated course participants guided by competent instructors within a programme employing simulated real-life scenarios played a crucial role in validating the effectiveness of the 4HM approach in IC and their readiness for change.

#### Harmonising practice: integrating 4HM into complex situations

In the third theme, participants targeted integrating 4HM principles into real healthcare situations to enhance patient and family outcomes.

Individuals stressed the critical role of managerial support and persistence during implementation. The course’s material needs ongoing reinforcement through departmental discussions using existing meeting platforms and engaging resource persons. Some emphasised the necessity for managers to enquire and offer guidance, providing clear direction for a task. Furthermore, participants emphasised the importance of psychological safety within the department, allowing space for mistakes and encouraging group reflection, thereby nurturing a culture of continuous improvement.

‘*We should utilise information platforms more to enhance learning retention. Although I’ve integrated these habits into my practice*,* the demands of our busy workday sometimes lead to unexpected task switches. While knowledge is present*,* it could be reinforced. Periodic reflection meetings could assist us in revisiting and contemplating what we’ve learned’.* (No. 15, focus group interview 2)

Some informants detailed obstacles to fully implementing the 4HM in IC interactions, citing complexities in patient cases, staff dynamics and organisational structures. Challenges included applying the 4HM to dementia patients, language and cultural barriers and frustrations in understanding patient perspectives amid high turnover and sudden room changes disrupting communication.

‘*We often have different patients*,* so one day I might have the first three rooms*,* and the next day*,* I could have completely different ones. I might have started something I intended to finish*,* but then I won’t have those rooms tomorrow. It’s like this all the time*,* fluctuating a lot. So*,* it’s not certain that the next person to come in will have the same focus as I did*’. (No. 4, focus group interview 1)

Despite encountering obstacles in their busy daily schedules, most participants successfully implemented the 4HM in numerous instances. They described this as adapting to the situation by integrating aspects of the 4HM. Participants emphasised the importance of engaging patients to cultivate a sense of security. They found the 4HM a valuable framework for facilitating effective dialogue. Importantly, applying 4HM did not extend interactions unnecessarily; rather, it saved resources by preventing frustration, and many participants reported positive outcomes from using the 4HM in their interactions with relatives.

‘*It’s a valuable insight that 4HM can be applied to relatives as well. They may require similar support to the patient. By investing time and effort in showing presence and empathy*,* whether at the beginning or during the process*,* staff may reduce the need for “firefighting” interventions later*’. (No. 7, focus group interview 2)

In essence, the successful incorporation of new routines such as 4HM requires supportive structures and management that prioritise communication and highlight opportunities, even in complex situations.

#### Embedding value: normalising the 4HM in everyday work

In the final theme, we emphasise 4HM’s value in IC, highlighting its effectiveness in improving patient care and becoming a standard practice, laying the foundation for potential expansion.

The participants collectively agreed that communication significantly impacts patient satisfaction and care quality, serving as a means of knowledge transfer and reassurance. However, some noted a lack of guidance on effectively involving patients despite recognising its importance. Specifically, course participants viewed the 4HM as a concrete tool for managing unexpected and challenging situations, offering better staff preparation.

‘*I’ve discovered that I can often resolve tense patient or relative situations by acknowledging concerns and offering reassurance. Using phrases we learned like “I understand” or “it’s good you’re speaking up” helps calm patients or relatives*,* leaving them satisfied with the interaction*’. (No. 11, focus group interview 1)

The course participants noted several changes in their communication habits following the 4HM course programme. First, they became more mindful of the importance of building a relationship with the patient, particularly during initial encounters, and recognising the value of sitting down with them. Second, they heightened their awareness of exploring the patient’s perspective and addressing challenging emotions. Third, they became more attentive to their own tone and body language. Finally, they developed an understanding of the significance of concluding interactions properly by asking, ‘Is there anything else you’re wondering about before I leave?’ to avoid the ‘doorknob syndrome’. Overall, they described integrating communication more extensively into their daily practices.

‘*I noticed that I’m more focused on the first habit*,* investing in the beginning*,* aiming to build a strong rapport and connection right from the start. Additionally*,* I prioritise habit four*,* ensuring a proper conclusion after each interaction. I always ask*,* “Is there anything else you need?” to ensure clarity and finalise our plans. This approach brings a sense of organisation to my interactions*’. (No. 5, focus group interview 2)

Course participants emphasised broader attendance to establish 4HM as standard practice, expressing concern for sustainability and stressing ongoing activities for continuity. Echoing these concerns, managers plan to train 4HM instructors across all IC institutions in Oslo and establish it as a regular course series, showcasing its utilisation within interdisciplinary teams and across care levels.

‘*This project is crucial*,* with feedback from employees confirming its importance. We’re ready to train all employees if we have instructors. It’s wise for the institution*,* itself*,* to manage it. We could designate a couple of individuals to take the instructor’s course*,* and our existing professional development days’ structure allows us to divide people into smaller groups for training*’. (No. 17, post-interview manager)

In summary, while participants have adapted their communication practices, it is imperative to train internal instructors to standardise the 4HM framework and facilitate its integration.

### Results from the NoMAD questionnaire

**Fig. 2:**
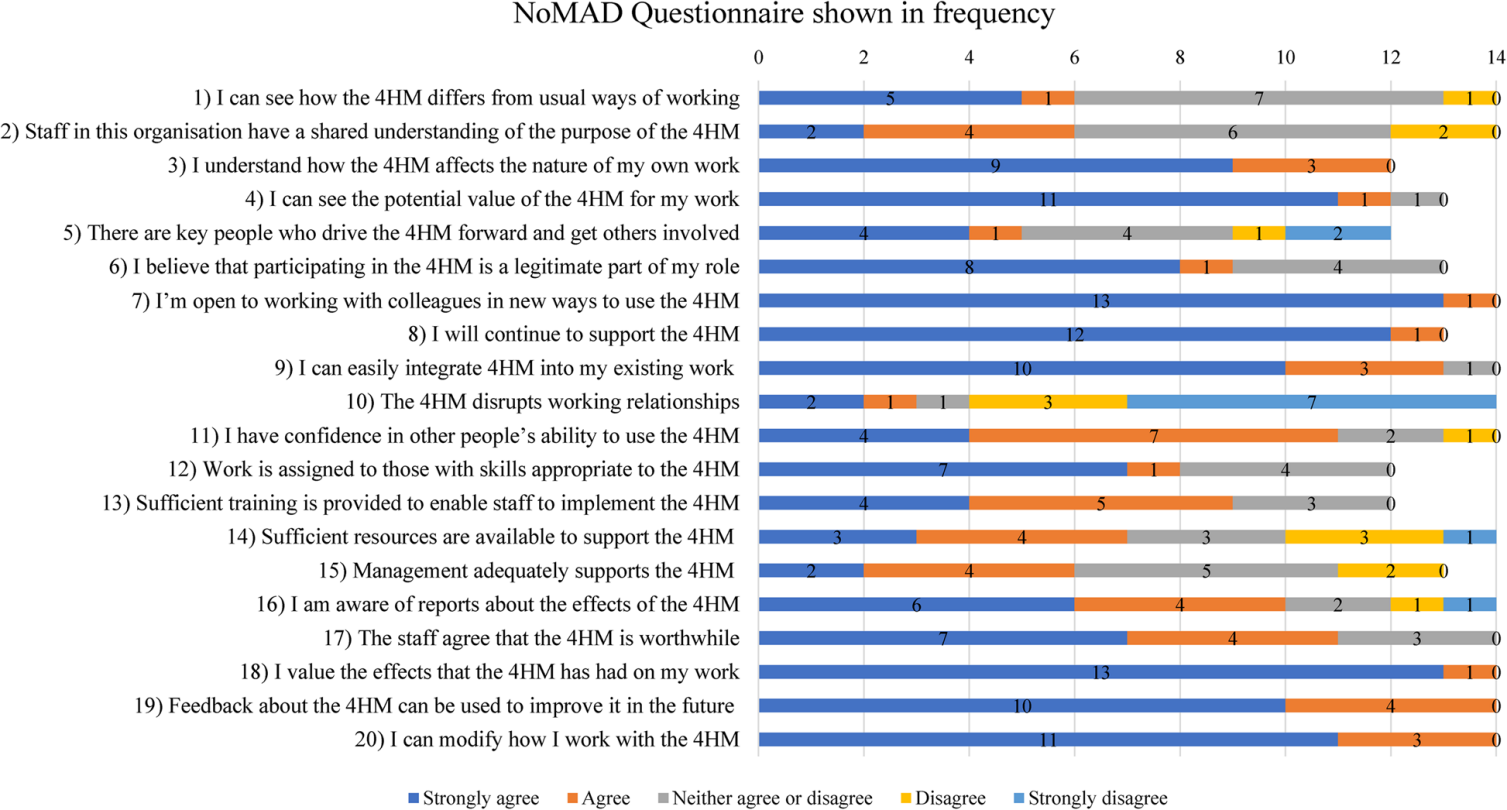
The NoMAD questionnaire assessed on a 5-point Likert scale (1=Strongly agree to 5=Strongly disagree)

Overall, the NoMAD questionnaire consistently indicated positive feedback on the 4HM implementation, with the highest scores for item 17: ‘I’m open to working with colleagues in new ways to use the 4HM’ and item 18: ‘I value the effects that the 4HM has had on my work’. Conversely, the items with the lowest scores were no. 5: ‘There are key people who drive the 4HM forward and get others involved’, no. 15: ‘Management adequately supports the 4HM’ and no. 2: ‘Staff in this organisation have a shared understanding of the purpose of the 4HM’. Most participants (*N* = 11) did not see the 4HM as disruptive (item 10); however, some struggled to differentiate it from existing practices (item 1, *N* = 8). Furthermore, after four months, the 4HM still felt familiar when utilised (median 8.5). In addition, the four habits are currently a normal part of their daily work (median 8.5) or at least will become (median 9), rated on a 1–10 scale, where higher scores indicated greater favourability towards normalisation. The course participants’ (*N* = 14) scores on the NoMAD questionnaire are illustrated in Fig. 2.

## Discussion

The first theme, ‘Decoding Interaction, Making Sense of the 4HM in IC Services’, corresponds to the NPT construct coherence and focuses on understanding *what* communication competence and the 4HM mean to healthcare professionals [37]. The 4HM course programme was deemed feasible for expansion within IC services as the progress in IC depends on strengthening communication and user involvement with leadership support crucial for anchoring the 4HM course programme. Both managers and staff found its focus sensible for addressing communication breakdowns and readiness for change. The NoMAD questionnaire confirmed the participants’ understanding of the importance of communication competence in IC and the relevance of the 4HM course.

Our results suggest that introducing the 4HM as an evidence-based practice rooted in high-quality research and contextualising it with real-life IC scenarios made the course programme meaningful and relevant for fully understanding of the 4HM. The importance of adapting and tailoring the intervention to the context is also highlighted as crucial by Proctor et al. (2013) for successful implementation. Adapting and tailoring interventions to local contexts is vital for their relevance, effectiveness, sustainability and earning communities’ respect [17]. The survey supports the qualitative analysis, indicating that while course participants appreciate the 4HM, some find it challenging to distinguish it from current practices. Furthermore, not all staff members possess a universal understanding of the 4HM, emphasising the need to extend the course to more staff members.

The second theme, ‘Fostering Change: Legitimising 4HM Through Staff Engagement’, aligns with the NPT construct of cognitive participation, and focuses on identifying *who* should be involved and ensuring their active particiation and engagement [37] Study participants strongly advocated inviting IC staff perceived as motivated to change, particularly resource persons who could serve as role models and sustain momentum. In the literature, motivated staff or resource persons are also termed *opinion leaders* [51]. According to Powell et al. (2015), opinion leaders play a crucial role in implementing strategies because they bring knowledge, help develop skills, solve problems and ensure the continuity of programmes through their connections. Opinion leaders hold significant influence and credibility, effectively shaping perceptions and driving change within their communities or networks. However, in the survey, a few respondents (*N* = 3) still expressed concerns about the absence of key persons advancing the 4HM. The course’s participants appreciated being relieved from clinical duties for two days to attend the 4HM course, which they viewed as a form of professional recognition. As emphasised by Powell et al. (2015), funding is crucial for implementation success. It provides the necessary resources, builds infrastructures and sustains operations – all essential for effective intervention implementation [51].

Individuals highlighted the vital role of trained instructors with clinical experience in fostering a supportive group atmosphere. Their ability to empathise with staff, comprehend their demanding work schedules and identify clinically relevant practice scenarios was highlighted. In our study, instructors with IC experience underwent Train-The-Trainer (TTT) education. This approach is recommended for IC institutions aiming to train their instructors for broader implementation. A recent systematic review highlighted the effectiveness of TTT programmes for healthcare professionals in disseminating knowledge. This supports the model’s assumption that local healthcare professionals can train their colleagues [52].

In our study, participants universally found simulation valuable for refining communication skills, especially when customised to real-life situations they selected, despite some finding it challenging and pushing their limits. Education emerged as the most used knowledge translation strategy to sustain evidence-based interventions in healthcare, according to a recent review [53]. Simulation entails training sessions mimicking real-life interactions between patients and staff, often through activities such as role playing [45]. Fidelity in simulation refers to how accurately it replicates real events or settings, including physical, psychological and environmental elements [54]. The 4HM courses utilise high-fidelity simulation techniques, providing staff with realistic interactive training experiences [54, 55].

The third theme, ‘Harmonising Practice: Integrating 4HM into Complex Situations’, aligns with the construct of collective action in NPT and explores *how* and in what circumstances healthcare professionals will apply the 4HM in IC [37]. Corresponding to our findings, all participants stressed the crucial role of managerial support and perseverance during implementation. They advocated leaders, in order to truly empower their teams, should remain visible, routinely inquire about progress, and actively make room for 4HM initiatives. However, according to the NoMAD, only six participants considered this support adequate. Leadership is highlighted as a key implementation strategy, with managers’ involvement shaping healthcare professionals’ priorities during implementation [17]. Their responsibilities for healthcare professionals, operations and budgets dictate resource allocation, including additional time, thereby shaping and guiding the implementation process [17, 56].

Both the focus group and individual interviews and the NoMAD questionnaire revealed that course participants noticed better communication after the course, applying the 4HM in various clinical scenarios. The participants found the 4HM to be universally applicable, not only in interaction with patients and families but also among team members. Yet, they faced structural barriers, such as turnover of temporary staff not fluent in Norwegian and challenges in communication with frail patients in a standardised system with high patient turnover. These results align with a recent meta-ethnography highlighting the challenges in implementing person-centred care within a standardised transitional care system. In such systems, healthcare professionals often lack the flexibility and autonomy needed to effectively address diverse patient needs [5].

The fourth theme, ‘Embedding value: Normalising the 4HM in everyday work’, aligns with reflexive monitoring in NPT. It captures the perceived values of the 4HM and investigates *why* it is incorporated into daily practices [37]. Participants agreed that effective communication, improved by IC staff after completing the 4HM course, enhances patient satisfaction and reduces complaints. In our study, both course participants and managers agreed that having few attendees to the 4HM course programme hindered its integration into practice. To make it a standard, leader prioritisation and ongoing discussions about the 4HM are vital. Flynn et al. (2023) highlighted a similar issue, in which the implementation and sustainability phases are perceived as distinct time periods. Our findings suggest that it is crucial to consider all implementation phases, including sustainability, as part of a continuous process. Therefore, when introducing new routines like the 4HM, we should design and select implementation strategies with this continuum in mind [53]. The survey confirms changes in communication and identifies areas needing attention, such as involving key individuals and securing managerial support.

According to the final manager interviews, implementing the 4HM in IC services requires training local instructors at each institution. In the final phase, to promote sustainability and normalisation, IC institutions will actively seek funding to support TTT education, ensuring the ongoing training of local instructors. They will embed the 4HM into their practice culture, reinforcing it during professional development and other platforms for consistency across wards. As illustrated by Jørgensen et al. (2019), five key domains are essential for successful implementation: patient perspectives, multidisciplinary team composition, bottom-up initiatives and skill building, managerial support and information sharing with colleagues – all of which we have addressed in our study [57].

### Strengths and limitations

To our knowledge, this is the first study to investigate 4HM course implementation in IC using the NPT. This study’s strengths lie in its process evaluation and the systematic use of strategies to accelerate implementation. To ensure trustworthiness, we conducted three assessments – pre-course, post-course and after four months – using several detailed methods. Open-ended questions were used in the interviews to establish trust. Emphasising a need-led approach rooted in a JLA process, this project involved an interdisciplinary research team (two physiotherapists, one medical doctor and one sociologist), all with clinical expertise and research experience. Recent reviews underscore the value of NPT in crafting interventions, devising implementation strategies and grasping the intricacies of the implementation process [39–41]. The number of participants was guided by Malterud’s information power, considering the study aim, participant specificity, interview quality, theoretical framework, and analytical approach [46]. This study adheres to Standards for Reporting Implementation Studies (StaRI) [58] and Standards for Reporting Qualitative Research (SRQR) [59].

This study has several limitations. While NPT enhances focus and rigour, it may limit enquiry, potentially overlooking relevant aspects. The inclusion of volunteers and motivated staff in the courses may have enhanced results. The first author’s extensive clinical experience in IC signals long-term engagement. To complement this, other authors provided analytical distance, scrutinising potential researcher biases. The NoMAD questionnaire analysis supplements qualitative findings. Despite confidence in qualitative trustworthiness, caution is advised in generalising due to the survey’s limited sample. Future research on a broader scale is recommended for a comprehensive examination.

## Conclusion

The 4HM course programme was found acceptable, appropriate, and feasible for expansion within IC services. Healthcare professionals and managers responsible for implementing new routines found the courses sensible, addressing communication breakdowns and fostering the readiness for change. Prioritising motivated participants, guided by skilled instructors using simulated scenarios, validated the 4HM approach in preparing for change. Successful integration merits supportive organisational structures and management, prioritising communication even in complex situations. Sustained integration entails systematically training internal instructors in the 4HM framework. This study’s findings are valuable for stakeholders implementing new routines in IC services, potentially enhancing service quality for older patients and their relatives along the clinical pathway.

## Supplementary Information

Supplementary Material 1

Supplementary Material 2

Supplementary Material 3

## Data Availability

As the data collection approval for the main study states the data to be available only to the researchers, data and materials collected for this manuscript will not be shared.

## Abbreviations

4HM: Four Habits Model
JLA: James Lind Alliance
IC: Intermediate Care
NoMAD: Normalization MeAsure Development
NPT: Normalisation Process Theory
TTT: Train-The-Trainer
