# Supplementary material for: Implementation of the four habits model in intermediate care services in Norway: a process evaluation
Source: BMC Health Serv Res. 2024 Oct 8;24:1196. doi: 10.1186/s12913-024-11647-z (PMC11460008; doi:10.1186/s12913-024-11647-z)
Supplement: Supplementary file 1 — Supplementary Material 1 [file 12913_2024_11647_MOESM1_ESM.docx]

**Interview guide for the pre-course individual interviews**

Can you please tell me a bit about your role here at the IC institution?

1. **Knowledge about user involvement**

- Could you please describe how the staff in IC prioritize user involvement?
- Does this focus involve awareness, knowledge, or perhaps both? Please explain.
- To what extent are the staff familiar with the benefits of user involvement?
- To what extent is user involvement associated with effective communication?

1. **Measures and leadership commitment to user involvement**

- To what extent have measures been implemented to promote user involvement in IC?
- In what way do you/and other staff here use the "What matters to you?" question?
- To what extent do you believe user involvement is endorsed by the management?
- What are your thoughts on inviting department managers to the course programme?

1. **Climate in departments regarding willingness to change**

- How do you perceive the current attitude towards improving user involvement?
- How do you think the staff would receive a communication course like the 4HM?
- Do you believe they will see the value of the course, and how will they show this?
- How can we, as instructors, foster a broad understanding of the course?

1. **Conducting the "Four Good Habits" course programme**

- How do you think we can best frame the "Four Good Habits" courses?
- Who should participate in the Four Good Habits courses in your opinion?
- What potential barriers do you see in using simulation as a method?
- How can we, as instructors, contribute to creating a safe atmosphere?

1. **Aspects related to the further integration of new practices**

- How can we ensure that the four good habits are discussed in clinical practice?
- How “ready” do you believe the staff in IC are for us to come and conduct the courses?
- Do you see any differences between departments that we should be aware of?
- What, in your opinion, is necessary to achieve lasting changes in practice?

1. **Closing and summarising the interview**

- Is there anything else you would like to share before we conclude the interview?
