# Supplementary material for: Implementation of the four habits model in intermediate care services in Norway: a process evaluation
Source: BMC Health Serv Res. 2024 Oct 8;24:1196. doi: 10.1186/s12913-024-11647-z (PMC11460008; doi:10.1186/s12913-024-11647-z)
Supplement: Supplementary file 2 — Supplementary Material 2 [file 12913_2024_11647_MOESM2_ESM.docx]

**Focus group interview with course participants**

1. **The experience of participating in the 4HM communication course**

- In general, how did you experience taking part in the 4HM course programme?
- Please share your thoughts on the various habits: 1) Invest in the beginning, 2) Elicit the patient’s perspective, 3) Demonstrate empathy, and 4) Invest in the end.
- What was your perception of the value derived from: i) the plenary sessions for each habit, ii) the instructors’ demonstration of role-playing, and iii) the engagement in role-playing as practitioner and patient/family member?

1. **The perceived utility of the 4HM communication course programme**

- Could you elaborate on the practical value and what you learned from the course?
- In what way have you altered your communication as a consequence of the course?
- How strongly do you link patient participation with effective communication and why?
- How has the course heightened your awareness of effective communication?

1. **Applicability of the 4HM communication course in the context of IC**

- How do you perceive the relevance of the 4HM communication course in the IC context?
- In which particular scenarios could the 4HM be beneficial in clinical practice?
- How might the remaining staff in IC gain advantages from this course?
- What do you think would be the outcome of implementing the 4HM in IC?

1. **Normalisation (implementation) of the 4HM into daily routine**

- How would you describe the 4HM compared to your previous communication methods?
- Is there a shared understanding of the practical value linked to the 4HM?
- How adequately have you been trained to utilise the 4HM?
- How much time and resources are available for the practical application of the habits?
- How thoroughly are the 4HM integrated into the leadership approach within IC?
- How do you plan to collaborate in implementing the four good habits in the future?

1. **Closing and summarising the interview**

- Is there anything else you would like to share before we conclude this group discussion?

**Individual interview guide for managers**

1. **Completion of the 4HM communication course programme**

- As a leader, how do you perceive the reception of the 4HM course?
- Please share your thoughts on each of the habits: 1) Invest in the beginning, 2) Elicit the patient’s perspective, 3) Demonstrate empathy, and 4) Invest in the end.
- What was your perception of the value derived from: i) the plenary sessions for each habit, ii) the instructors’ demonstration of role-playing, and iii) the engagement in role-playing as practitioner and patient/family member?

1. **Utility of the 4HM communication course**

- Could you elaborate on the course’s practical value and what HCPs might have learned?
- In what way do you believe HCPs have altered their communication post-course?
- How strongly do you link patient participation with effective communication and why?
- How has the course heightened your awareness of effective communication?

1. **Applicability of the 4HM communication course in the context of IC**

- As a leader, how do you perceive the relevance of the 4HM in the IC context?
- In which particular scenarios could the 4HM be beneficial in clinical practice?
- How might the remaining staff in IC gain advantages from this course?
- What do you think would be the outcome of implementing the 4HM in IC?

1. **Normalisation (implementation) of the 4HM into daily routine**

- How would you describe the 4HM compared to today’s communication methods?
- Is there a shared understanding of the practical value linked to the 4HM?
- How adequately have HCPs been trained to utilise the 4HM?
- How much time and resources are available for the practical application of the habits?
- How thoroughly are the 4HM integrated into the leadership approach within IC?
- As a leader, how do you plan to collaborate in implementing the 4HM in the future?

1. **Closing and summarising the interview**

- Is there anything else you would like to share before we conclude this interview?
