# Supplementary material for: Implementation of the four habits model in intermediate care services in Norway: a process evaluation
Source: BMC Health Serv Res. 2024 Oct 8;24:1196. doi: 10.1186/s12913-024-11647-z (PMC11460008; doi:10.1186/s12913-024-11647-z)
Supplement: Supplementary file 3 — Supplementary Material 3 [file 12913_2024_11647_MOESM3_ESM.docx]

**The NoMAD questionnaire – four months post-course**

Background information:

IC institution: ______________ Sex: ________ Profession: __________________

Department: _______________ Age: ________ Educated (year): _____________

How many years have you been working in the IC services? (Check the appropriate box)

| Less than 1 year |  | 2 – 5 years |  | More than 5 years |  |
| --- | --- | --- | --- | --- | --- |

On a scale from 1 to 10 (please circle the correct number)

| **When you use the 4HM, how familiar does it feel?** |
| --- |

**Still feels very new Feels completely familiar**

| **1** | **2** | **3** | **4** | **5** | **6** | **7** | **8** | **9** | **10** |
| --- | --- | --- | --- | --- | --- | --- | --- | --- | --- |

| **Do you feel the 4HM is currently a normal part of your work?** |
| --- |

**Not at all Somewhat Completely**

| **1** | **2** | **3** | **4** | **5** | **6** | **7** | **8** | **9** | **10** |
| --- | --- | --- | --- | --- | --- | --- | --- | --- | --- |

| **Do you feel the 4HM will become a normal part of your work?** |
| --- |

**Not at all Somewhat Completely**

| **1** | **2** | **3** | **4** | **5** | **6** | **7** | **8** | **9** | **10** |
| --- | --- | --- | --- | --- | --- | --- | --- | --- | --- |

**For each statement please select an answer that best suits your experience**

|  | Strongly agree | Agree | Neither agree nor disagree | Disagree | Strongly disagree |
| --- | --- | --- | --- | --- | --- |
| I can see how the 4HM differs from usual ways of working |  |  |  |  |  |
| Staff in this organisation have a shared understanding of the purpose of the 4HM |  |  |  |  |  |
| I understand how the 4HM affects the nature of my own work |  |  |  |  |  |
| I can see the potential value of the 4HM for my work |  |  |  |  |  |
| There are key people who drive the 4HM forward and get others involved |  |  |  |  |  |
| I believe that participating in the 4HM is a legitimate part of my role |  |  |  |  |  |
| I’m open to working with colleagues in new ways to use the 4HM |  |  |  |  |  |
| I will continue to support the 4HM |  |  |  |  |  |
| I can easily integrate the 4HM into my existing work |  |  |  |  |  |
| The 4HM disrupts working relationships |  |  |  |  |  |
| I have confidence in other people’s ability to use the 4HM |  |  |  |  |  |
| Work is assigned to those with skills appropriate to the 4HM |  |  |  |  |  |
| Sufficient training is provided to enable staff to implement the 4HM |  |  |  |  |  |
| Sufficient resources are available to support the 4HM |  |  |  |  |  |
| Management adequately supports the 4HM |  |  |  |  |  |
| I am aware of reports about the effects of the 4HM |  |  |  |  |  |
| The staff agree that the 4HM is worthwhile |  |  |  |  |  |
| I value the effects that the 4HM has had on my work |  |  |  |  |  |
| Feedback about the 4HM can be used to improve it in the future |  |  |  |  |  |
| I can modify how I work with the 4HM |  |  |  |  |  |
